# Towards a Unified Neural Mechanism for Reactive Adaptive Behaviour

**DOI:** 10.1016/j.pneurobio.2021.102115

**Published:** 2021-06-24

**Authors:** Giacomo Novembre, Gian Domenico Iannetti

**Affiliations:** 1 Neuroscience and Behaviour Laboratory, Istituto Italiano di Tecnologia (IIT), Rome, Italy; 2 Department of Neuroscience, Physiology and Pharmacology, University College London, UK

**Keywords:** Electromyography (EMG), Electroencephalography (EEG), Movement, Sensorimotor processing, Saliency, Surprise

## Abstract

Surviving in natural environments requires animals to sense sudden events and swiftly adapt behaviour accordingly. The study of such Reactive Adaptive Behaviour (RAB) has been central to a number of research streams, all orbiting around movement science but progressing in parallel, with little cross-field fertilization. We provide a concise review of these research streams, independently describing four types of RAB: (i) cortico-muscular resonance, (ii) stimulus locked response, (iii) online motor correction and (iv) action stopping. We highlight remarkable similarities across these four RABs, suggesting that they might be subserved by the same neural mechanism, and propose directions for future research on this topic.

## Introduction

In the animal world, movement and life go hand in hand: an animal not able to move effectively is less likely to survive. Yet, the constraints posed by living in a rapidly-changing environment have pushed brains to evolve not only a sophisticated motor system, but also a tight coupling between movement and sensory encoding. Development of the motor system is indeed guided by perception. Likewise, perception alone does not develop properly without movement (1).

Out of the myriad of examples of sensory-motor integrative processes only some are subject to volition – the individual’s ability to choose whether or not to act in a given circumstance (2). For instance, a monkey actively looking for food might deliberately choose to climb a particular tree when it sees lots of fruits hanging on it. Yet, a sudden and unexpected change in the sensory scene might trigger an unavoidable reactive behaviour, having higher priority compared to the initial goal to collect food. A rustling coming from the branches above the monkey might lead the animal to jump out of the tree. The same sound coming from the ground might instead lead the monkey to climb even higher. Either of these clearly distinct actions – climbing or jumping off the tree – albeit appropriately chosen on the basis of the context, take often place with none or scarce influence of the animal’s volition. Yet, either is indispensable for survival.

Here we refer to this way of acting (or modulation of acting) as “Reactive Adaptive Behaviour” (RAB). RAB falls in the nexus between reflexive and volitional movements. Similarly to reflexes, RAB is reactive and therefore stimulus-driven. However, similarly to voluntary actions, RAB is adaptive, i.e. flexible to the ever-changing nature of the environment. As such, RAB questions the very dichotomy between reflexes and voluntary actions (3, 4), calling for a reflexive-volitional gradient wherein RAB itself lies.

We highlight four fundamental features that apply to RAB. First, RAB is evoked by sudden and unexpected changes in the sensory scene, i.e. by “salient” stimuli. Second, RAB is characterized by its short-latency: it is rapid, and elicited in situations where speedy responses can be vital. Third, RAB is adaptive, i.e. flexible on the basis of the context: it favours those behaviours (e.g. climbing vs. jumping, according to the above example) that ensure survival and, in the long term, maximise fitness. This implies that RAB is the result of relatively complex neural computations, selecting motor output on the basis of the current environmental context. Fourth, as already anticipated above, RAB takes place with none or scarce influence of a subject’s volition.

Considering the above features, it turns out that RAB has been studied across a wide range of disciplines concerned with studying biological movement, such as neuroscience, psychology and biomechanics. Over the past couple of decades, a number of original observations have characterized specific manifestations of RAB using distinct experimental paradigms. Yet, we were stunned to realise that these research streams are largely progressing in parallel, with little cross-field fertilization. This prompted us to conceive the current work. We aim to provide a concise review of some research streams independently describing four particular RABs: (i) Cortico-Muscular Resonance, (ii) Stimulus Locked Response, (iii) Online Motor Correction and (iv) Action Stopping. While doing so, we highlight how each of these behaviours fulfils the above-described criteria for RAB, and discuss the possibility that they could be partly subserved by the same neural mechanism.

### [i] Cortico-Muscular Resonance (CMR)

The term Cortico-Muscular Resonance (CMR) has been recently proposed to refer to a series of fast modulations of muscular activity (and ensuing applied force) evoked by sudden sensory stimuli, irrespectively of their sensory modality (5, 6).

**Figure 1:**
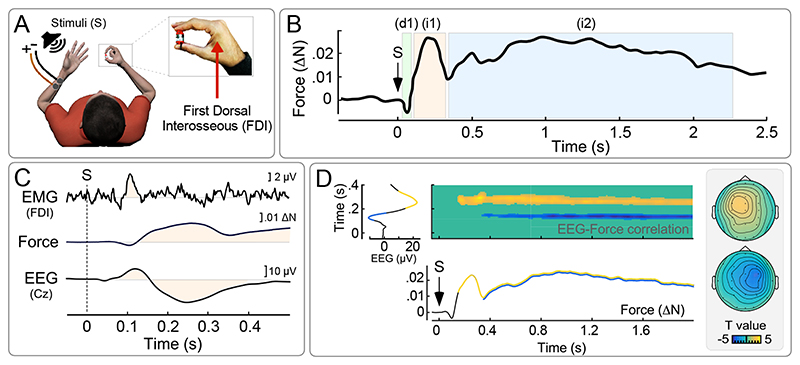
Cortico-Muscular Resonance (CMR). (A) Participants exert a weak and constant isometric force holding a transducer between the right index finger and thumb (~1 N). Both force and EMG (from the First Dorsal Interosseous, FDI) are simultaneously recorded. Somatosensory stimuli are delivered through electrical stimulation of the left median nerve or auditory stimuli are delivered through a loudspeaker placed nearby the left hand. (B) These fast-rising stimuli, regardless of sensory modality, elicit a multiphasic modulation of the exerted force, consisting of an initial decrease (d1, peaking ~100 ms post-stimulus), followed by a first transient increase (*i1*, peaking ~250 ms post-stimulus) and a second more tonic increase (*i2*, starting ~350 ms post-stimulus). (C) Simultaneous measurements of EMG activity (from FDI), Force, and EEG (at Cz): Signal modulations that co-vary across measurements are highlighted – see (5) for details. Time 0 indicates stimulus onset (S). Note that Force recordings lag behind EMG due to the well-known electromechanical delay of motion with respect to muscular activity (Box 1). (D) Trial-by-trial correlations between all timepoints of simultaneously collected EEG and Force modulations in response to the same somatosensory stimulation. Both *i1* and *i2* correlate with a widespread EEG positivity contralateral to the hand exerting the force (yellow, top scalpmap). Additionally, *i2* correlates with an EEG negativity contralateral to the stimulated hand (blue, bottom scalpmap). Adapted with permission from (5).

**Figure 2:**
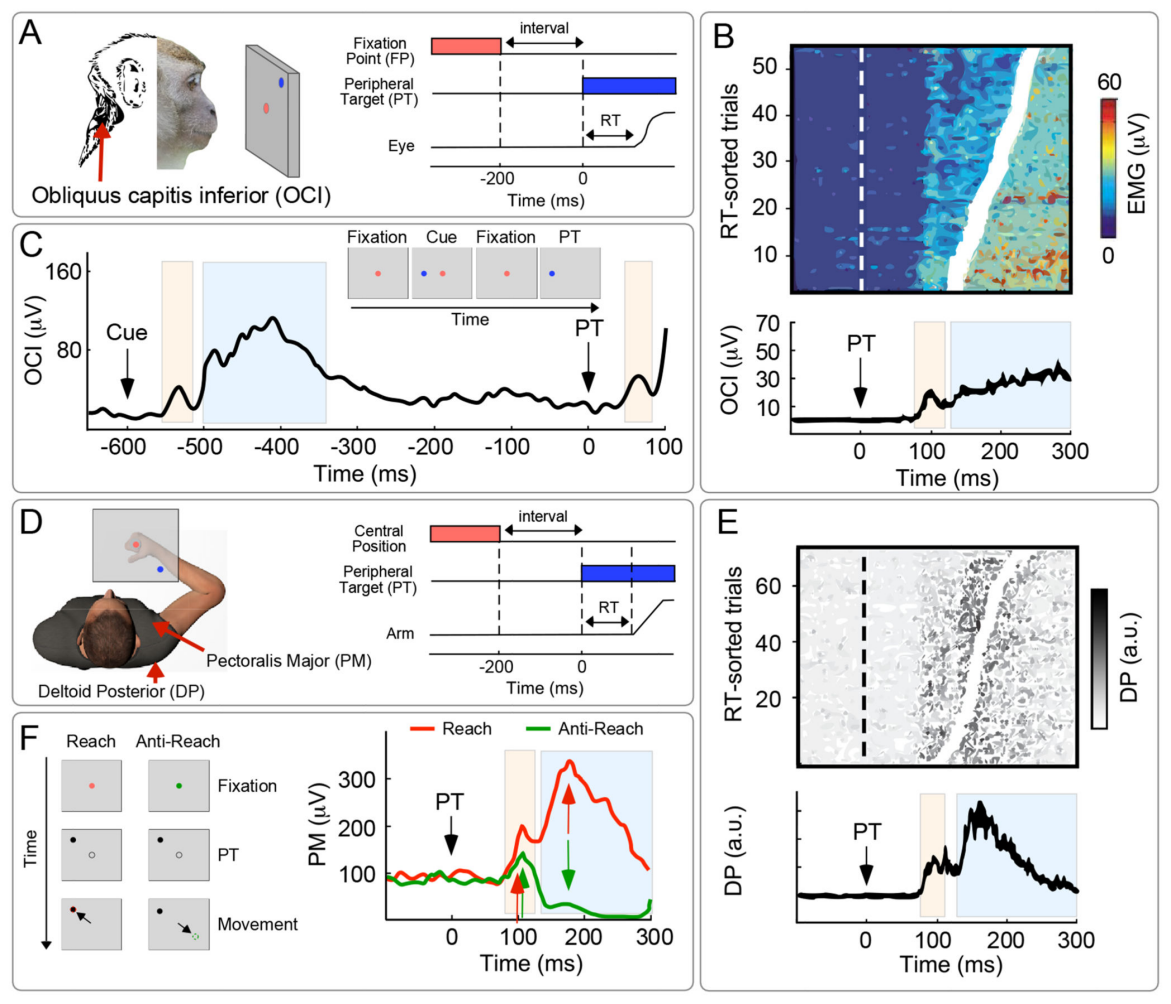
Stimulus Locked Response (SLR). (A) Paradigm used to study SLR in monkeys: The animals are trained to look at a central fixation point (FP). After a variable amount of time, the FP disappears, and the monkeys have to look to a new suddenly appearing peripheral target (PT). Intramuscular EMG activity is measured from several neck muscles including the obliquus capitis inferior (OCI), which subserves the rotation of the head towards the target. (B) The PT evokes in OCI a first transient burst of muscular activity (pink area), followed by a second period of tonic activity lasting until the saccade onset (light blue area). The magnitude of the transient burst predicted the latency of the following saccade. Adapted from (13). (C) Even when the animals do not need to produce a saccade (i.e. after the cue), the stimulus evokes the same pattern comprising two distinct phases. Adapted from (18). (D) Paradigm used to study SLR in humans: Participants move their arm under a non-transparent screen (shown in opaque in the figure for illustrative purposes), while only the visual feedback of the hand position is provided as a coloured dot. They are instructed to reach for the PT when this appears. Intramuscular EMG is recorded from a number of shoulder and arm muscles, including the deltoid posterior (DP) and the pectoralis major (PM). (E) PT appearance elicits a transient EMG burst (pink area), followed by the EMG activity consequent to the actual voluntary movement (light blue area). The magnitude of the first burst predicted the latency of the subsequent voluntary movement. Adapted from (12). (F) SLR implies a force useful for reaching the PT, even if participants are instructed to reach a location diametrically opposite to the PT. Adapted from (25).

CMR has been observed using both electromyography (EMG) and force measurements. In a typical experiment, participants are required to exert a weak and constant isometric force on a transducer held between the index finger and thumb, while simple, task-irrelevant and fast-rising sensory stimuli (either auditory or somatosensory) are delivered (5, 6) (Fig. 1a). These stimuli evoke a multiphasic modulation of the exerted force: An initial transient force decrease (*d1*), peaking ~100 ms post-stimulus, is followed by a transient force increase (*i1*) peaking at ~250 ms, and by a second (longer lasting) force increase (*i2*) starting ~300-350 ms and lasting for ~2 seconds (Fig 2b). The two initial force modulations – *d1* and *i1* – have an EMG counterpart, detected when recording from the first dorsal interosseous muscle (FDI; i.e., the muscle contributing to the force exerted on the transducer using the index finger, Fig 1a). Expectedly, the EMG modulations have shorter latencies (~75 and ~110 ms, respectively) compared to the corresponding force modulations, due to the well-known electromechanical delay of motion with respect to muscular activity (Fig. 1c, Box 1).

A few CMR features are worth being highlighted, as they nicely dovetail the features defining RAB (discussed in the previous section). First, CMR appears to be scarcely accessible to volition: not only participants are not meant to move in response to the stimuli, but they were mostly unaware of the modulation of their force output. Second, the CMR magnitude is considerably reduced when the eliciting stimulus has low behavioural relevance, e.g. when it is highly predictable (5). This observation highlights the adaptive character of the CMR, which is adjusted on the basis of the context, and preferentially triggered in response to stimuli that are more likely to require a swift reaction. Another feature that we highlight is that the CMR pattern evoked by auditory and somatosensory stimuli is extremely similar, indicating that CMR is consequent to a supra-modal neural mechanism. Notably, some research has described CMR-like modulations using also visual stimuli (7, 8).

The neural origin of CMR was explored using EEG recordings. It was observed that the stimuli eliciting CMR also evoke a concomitant Event Related Potential (ERP), dominated by two large negative-positive waves maximal at the scalp vertex (and therefore called ‘vertex potential’ (9, 10)) (Fig 1c). Like CMR, this ERP is evoked irrespectively of the modality of the stimulus, and its amplitude is reduced when the stimulus is highly expected (10, 11). Importantly, trial-by-trial analysis of simultaneous EEG-force recordings showed that the ERP and the CMR are tightly coupled: brain activity measured above the motor cortex contralateral to the hand exerting the force predicts the magnitude of *i1* and *i2*. Furthermore, brain activity measured contra-laterally to the hand receiving a somatosensory stimulus predicts the magnitude of *i2* (Fig. 1d). All together, this suggests that CMR originates from the effect of the saliency-induced vertex potential on the activity of specific cortical modules engaged in a certain task, including the corticospinal drive arising from frontal premotor/motor areas during the exertion of isometric force (5, 6).

### [ii] Stimulus Locked Response (SLR)

The term Stimulus Locked Response (SLR) has been coined to indicate short latency modulations of EMG activity evoked by sudden visual stimuli (12). These responses are typically recorded using intra-muscular EMG from neck and/or shoulder muscles of either human or non-human primates (12–14). SLRs exhibit a number of features typical of RAB, as discussed below.

#### [2.1] SLRs in non-human primates’ neck muscles during saccade tasks

SLRs were first observed by Corneil et al., (2004) in monkeys performing a saccade task (Fig. 2a) [(13) task adapted from (15)]. Animals were trained to look at a central fixation point (FP). After a variable amount of time, the FP would disappear, and the monkeys had to look to a suddenly-appearing new peripheral target (PT), presented in one of two diametrically opposite positions. The authors of this study noticed that, irrespective of whether the animals’ head had been restrained, three neck muscles that would turn the head towards the target (*obliquus capitis inferior, rectus capitis posterior maior* and *splenius capitis*) exhibited a first transient burst of muscular activity ~90 ms after PT appearance, and a second period of tonic muscular activity lasting until the saccade onset (Fig 2b). Notably, the latency of the first response was too short to be explained by a voluntary motor command and, most importantly, it was time-locked to stimulus presentation, and not to the ensuing saccade (which could be performed up to 150 ms following the first transient burst, and whose latency had a remarkably higher variability compared to that of the first burst). Suggesting a functional significance of this phenomenon, the magnitude of the transient burst predicted the latency of the following saccade, as if the neck musculature was ‘warming up’ while the decision to move was being formed (16, 17).

The SLR pattern of EMG activity comprising two consecutive responses, a transient burst followed by a more sustained enhancement (Fig 2b), is strongly reminiscent of the CMR, which also entails two consecutive force increases, the first being more transient and the second being more tonic (see section [2]; Fig 1b). Bearing in mind that CMR and SLR studies entailed different measures, stimulus modalities, tasks and species, the reader might wonder whether it is justified to suggest a relationship between the SLR and the CMR. It is difficult to answer this question, especially considering that the paradigm used in the first SLR investigation entailed a voluntary movement overlapping with the late parts of the CMR (13). However, in a following study, the same group used a cueing task (18). Briefly, monkeys were trained to saccade to a target, but before the target appearance a task-irrelevant cue was presented at either the same or the opposite location of the following target. When cue and target were separated by a sufficiently long time (i.e. 600 ms), it appeared that the cue alone evoked the same two EMG modulations in the head-turning neck muscle (*obliquus capitis inferior*) (Fig. 2c). Thus, even in the absence of a subsequent overt action, the cue evoked the typical multi-phasic SLR pattern, making its similarity with the CMR striking. We believe that this similarity is worth being explored in the future (BOX 2).

These SLRs (observable even before a overt action) were interpreted as suggestive of a “reflexive covert orienting” mechanism useful to “warm up” the neck musculature while the possible decision to presumably move the head and the eyes in synergy is formed (16, 17). This functional interpretation is not different from that provided for the CMR. However, when it comes to hypothesize the neural circuits underlying these responses, these interpretations differ considerably, at least on the surface. In fact, SLRs have been mostly interpreted as the result of a largely subcortical machinery, involving the tecto-reticulospinal pathway and the superior colliculus (17, 19). Instead, the CMR – as the name itself implies – appears to be related to activity of the cerebral cortex, and specifically the activity (or the modulation) of the motor cortex. These two accounts are, however, not mutually exclusive. Indeed, it has recently been suggested that the cortex might contribute to the early SLR, for instance by priming the putative subcortical circuit with information related to higher-level processing of the sensory input or contextual and task specific constrains (20–22). Hence, it is conceivable that SLR and CMR might be unified as being subserved by a single neural mechanism or network – a hypothesis obviously requiring careful scrutiny.

#### [2.2] SLRs in humans’ shoulder and arm muscles during reaching tasks

Following the first description in non-human primates, a number of studies have reported the existence of SLRs in humans (12, 23–26). Such studies have mostly adopted arm reaching tasks, often performed in the presence of a constant force field opposing the reach direction (27), given that a sustained background EMG activity appears to enhance the detectability of SLR (23). Again, the enhancement of the SLR in the presence of a stronger background force is reminiscent of the CMR, which is also optimally elicited during active isometric force exertion (5) and whose magnitude increases with enhanced background EMG activity (unpublished observation).

In the first SLR study in humans (12), participants could move their arm under a screen, while only the visual feedback of the hand position was provided as a coloured dot (Fig. 2d). Like in primate studies, participants had to hold the dot in a central position, and then move it to a peripheral target appearing in one among several possible locations. Intramuscular EMG was recorded from a number of shoulder (*deltoid posterior, pectoralis major*) and arm (*triceps lateral, brachioradialis*) muscles. The appearance of the peripheral target elicited a transient burst of muscle activity after approximately 100 ms (Fig. 2e). This initial burst of EMG activity was followed by the EMG activity consequent to the voluntary movement. Similarly to the SLR in primates, these EMG activities: (*i*) were spatially tuned, providing a glimpse of the pattern of muscle activation that would characterize the following voluntary action, and (*ii*) their amplitude predicted the overt response time latency.

SLRs fall nicely within the definition of RAB: they are evoked by fast-appearing stimuli, entail short-latency responses, and favour adaptive behaviour such as the preparation of an upcoming action in a spatially-tuned manner. A recent study (25) also examined whether the spatial tuning of the SLRs depends on volition – another feature defining RAB. The colour of a visual cue informed participants whether to perform a “reaching” movement towards a subsequently presented peripheral target, or an “anti-reaching” movement away from it (see also (28, 29), task adapted from (30)). This elegant design neatly dissociates the effects of stimulus position and goal position, which are congruent during “reaches” and incongruent during “anti-reaches” (Fig. 2f). The authors made an important observation: SLRs occurred in muscles necessary to move *towards* the target, irrespective of whether participants had to perform the reaching or anti-reaching movement (although, in the latter case, SLRs were slightly attenuated). In other words, the SLR implied the specification of a force useful for reaching the target, even though participants later voluntarily moved the arm to a diametrically opposite location. Thus, SLR depends mostly on the position of the suddenly-appearing visual target and can be only mildly modulated by volition.

### [iii] Online Motor Correction (OMC)

There is a third body of work investigating motor responses falling within the criteria that define RAB. This literature describes how sensory events cause adjustments during action execution. For example, while moving the arm to reach a cup, an unexpected event such as someone hitting your arm requires the movement to be corrected on the basis of proprioceptive and visual feedback. These adjustments are labelled Online Motor Correction (OMC). They are normally studied combining kinematic and EMG measures, and are assumed to be mediated by a number of cortical regions within the broad frontoparietal circuits that are often associated with goal-directed behaviour (3, 31–33). Like CMR and SLR, also OMC responses fulfil the criteria defining RAB.

The first studies of the neurophysiological processes underlying OMC date back to the middle of the last century. In the seminal work of Peter Hammond [(34, 35), reviewed in (36)], human subjects were required to exert a constant force to hold a weight attached to their wrist with a cable. A sudden perturbation – the pulling of the cable causing a stretch of the biceps muscle as well as displacement of the arm – evoked two EMG responses in the *ipsilateral biceps*: A first, short-latency monosynaptic response, peaking around 30 ms post stimulus (most likely reflecting a stretch reflex (37)), followed by a second polysynaptic response, occurring at 50-100 ms post stimulus. Notably, the second response was observed irrespectively of whether participants were instructed to “resist” or to “let go” the perturbation. This suggests that, like the previously reviewed CMR and SLR, this second polysynaptic response underling OMC is also weakly modulated by volition (but see more direct evidence below).

Later studies considerably enriched our understanding of the second polysynaptic response described by Hammond. We now know that this was likely the summation of (at least) two independent responses called R2 and R3, peaking ~60 and ~90ms post perturbation, and reflecting two distinct phases of a hierarchical OMC process (3, 38). Specifically, it has been proposed that R2 mostly reflects correction of “how” to achieve a given goal, such as which trajectory employing while reaching for a cup of coffee. Instead, R3 would mostly reflect correction of “what” goal to achieve (e.g. reaching for a cup of coffee vs. a different object nearby) (3, 39, 40).

**Figure 3:**
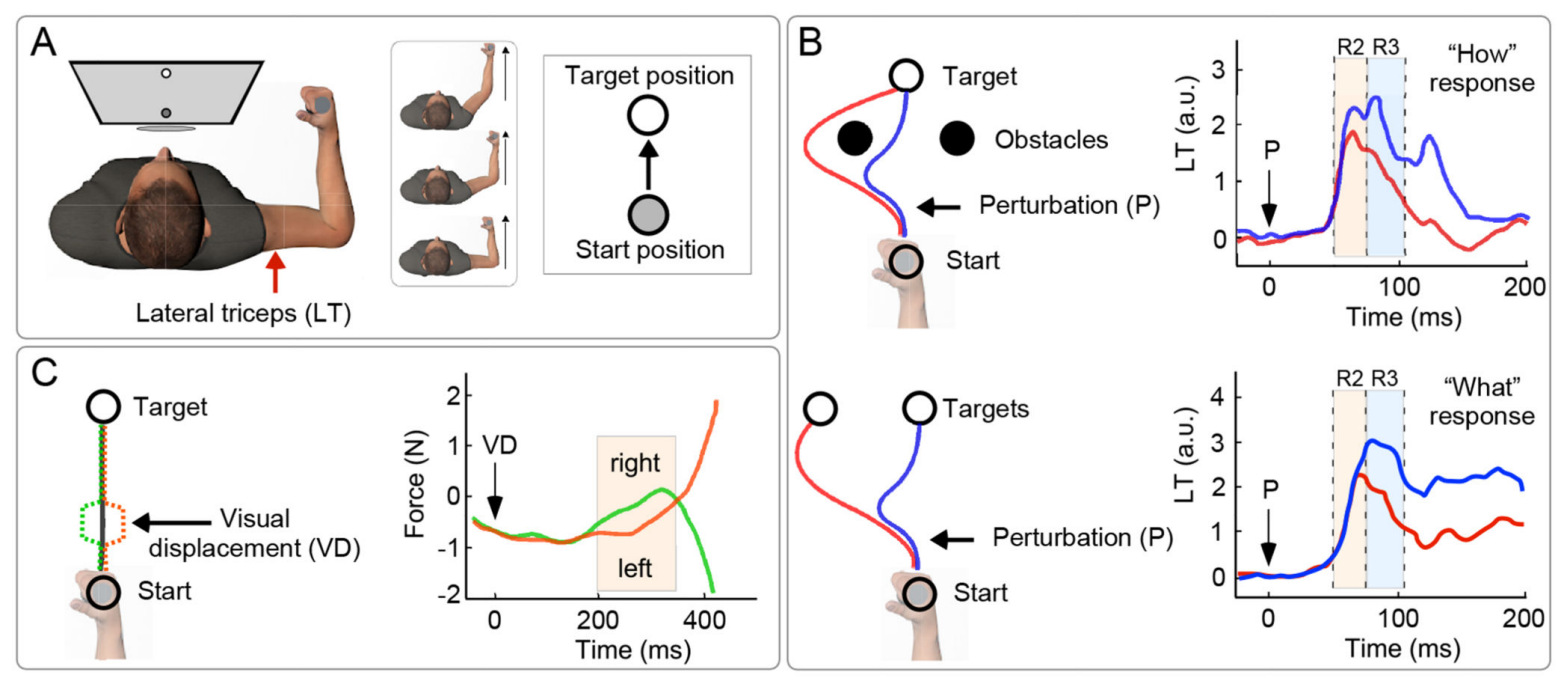
Online Motor Correction (OMC). (A) Participants are instructed to make an arm movement to reach a target with their hand (on a bi-dimensional plane) while a force was applied to activate their elbow extensor (lateral triceps, LT), which was recorded using EMG. Either a mechanical perturbation (P; panel b) or a visual displacement (VD; panel c) of the hand is used to trigger an OMC of the ongoing arm trajectory. (B) Participants receive a mechanical perturbation displacing their hand (black arrow). Top: If the correction implies a change of route in order to reach the goal (‘how’ change), an R2 is observed in LT ~60 ms post-perturbation. Bottom: If the correction implies a change of target to be reached (‘what’ change; note that after the perturbation the subject is allowed to choose whether hitting target A or B), an R3 is observed ~90 ms post-perturbation (displayed signals are obtained after subtracting the activity of unperturbed trials). Adapted from (39). (C) If participants are instructed to perform a voluntary movement in the same direction of a visual perturbation (VD in the right graph indicates the displacement onset at time = 0), an OMC identical to that observed without instruction (i.e. in the direction opposite to the perturbation) is observed, followed by the voluntary response in the same direction of the perturbation. Adapted from (43).

One elegant example of such functional dissociation was provided by Nashed et al. (39). Participants were instructed to reach a target with their arm (on a bi-dimensional plane) while a force was applied to activate their elbow extensor (*lateral triceps*), whose activity was recorded using EMG (Fig. 3a). Notably, participants were instructed to perform the task while avoiding two obstacles placed in between the start and the end position (Fig. 3b, top). On some trials, participants received a somatosensory perturbation that displaced their arm so that it would be likely to hit one of the two obstacles – hence requiring an OMC. It was observed that such OMC was adaptive, i.e. it was different depending on how far the perturbation had displaced the arm from the original pathway (note that the size of the arm displacement caused by the perturbation depended on small deviations in the arm trajectory prior to the perturbation, and not on the magnitude of the perturbation). In particular, following a large displacement of the arm, participants revised their pathway to circumvent the obstacles, while following a small displacement of the arm they stuck to the original pathway and reached the goal by passing between the obstacles. Such “optimized” correction – adaptively minimizing path length in a context-dependent manner – was underlined by a muscular burst observable ~60ms following the perturbation, i.e. during the R2 epoch (Fig. 3b, top).

A complementary experiment tested the effect of a perturbation that did not prompt participants to change “how” to reach the target, but “which” target to reach (Fig. 3b, bottom) (39). Specifically, following the arm displacement, participants could freely choose whether to reach for the original target or for another target placed nearby to where the arm had been displaced. In this case the OMC, implying a revision of the movement goal, occurred ~90 ms following the perturbation, i.e. during the R3 time window (Fig. 3b, bottom).

Similarly to the CMR, OMCs are also supra-modal: they occur not only following somatosensory perturbations but also in reaction to sudden changes in the visual environment (41, 42). Also in the visual domain it is possible to distinguish between corrections of “how” to achieve a given goal, and “what” goal to achieve. For instance, coming back to the previous example of a hand reaching for a cup, a sudden change of the perceived hand position (consequent to a surreptitiously altered visual feedback of the hand position) would imply a correction of how to achieve a goal, while a change of position of the cup would imply a correction of which goal to achieve (8, 43). Notably, these two distinct changes evoke OMC occurring after ~90 ms (R2: “how” correction) and ~110 ms (R3: “what” correction), respectively (8). Given the longer processing time of visual input compared to proprioceptive input (Box 1), this result is reminiscent of the previously discussed hierarchical organization of R2 and R3 elicited by somatosensory stimuli, and of their functional significance [(3), but see (44)].

Another RAB-like feature of OMC – aligning it with both CMR and SLR – is that it is weakly modulated by volition. Evidence comes from a study by Franklin and Wolpert where participants moved their arm, visualized as a cursor, from a start to an end target (43). The cursor position was suddenly displaced (1.5 cm, orthogonally to the reaching direction) from the current hand position, and then swiftly restored to the actual hand position (the cursor displacement lasted 230 ms in total, see Fig. 3c). An OMC was observed in the EMG (*pectoralis major*), at ~100 ms following the onset of the cursor displacement, and in the force, at ~150 ms (BOX 1). To determine whether this OMC was voluntary or not, participants were instructed to perform a voluntary movement in the same direction of the perturbation, i.e. opposite to the OMC. Remarkably, even in this condition, there was an OMC identical to that observed in the experiment without instruction (i.e. in the direction opposite to the perturbation), followed by the voluntary response in the same direction of the perturbation (Fig. 3c). Thus, this experiment elegantly demonstrated that sensory-driven OMC are not voluntarily generated, but are largely automatic responses, poorly modulated by volition^1^.

### [iv] Action Stopping (AS)

Another scientific community (and related literature) investigates RAB by examining the interruption of an ongoing action following a sudden stimulus, which is labelled Action Stopping (AS). This phenomenon is reminiscent of OMC in that it entails a change of ongoing motor behaviour. However, here the emphasis is placed on the *interruption*, not on the correction. For instance, if someone hits your arm while you are reaching for an object in the dark, you might correct the movement trajectory (as in OMC), or you might stop the reaching movement entirely (as in AS). Under the assumption that AS is meant to prevent a future error (following the example above, not reaching the object), AS is often considered an adaptive behaviour (45, 46).

In the laboratory, AS is normally studied using the Stop Signal Task (SST) (47, 48). In the classic version of the SST, a “go signal” instructs a participant to perform an action such as pressing a button. After the action has been initiated, in a minority of trials, a sudden “stop signal” instructs the participant to interrupt the ongoing action. Depending on the time interval between the go signal and the stop signal, the action might or might not be successfully stopped. This allows the estimation of the following parameters: (i) the probability of stopping as a function of go-stop time interval, (ii) the response time of “go trials” (i.e. trials without a stop signal), and (iii) the response time of unsuccessfully stopped trials (which, notably, exhibits faster RTs compared to go trials). This information is used to compute what authors in this field call “stop signal reaction time”: a value, ranging between 200 and 300 ms, indexing how long it takes to voluntarily cease an ongoing action (47, 49).

**Figure 4:**
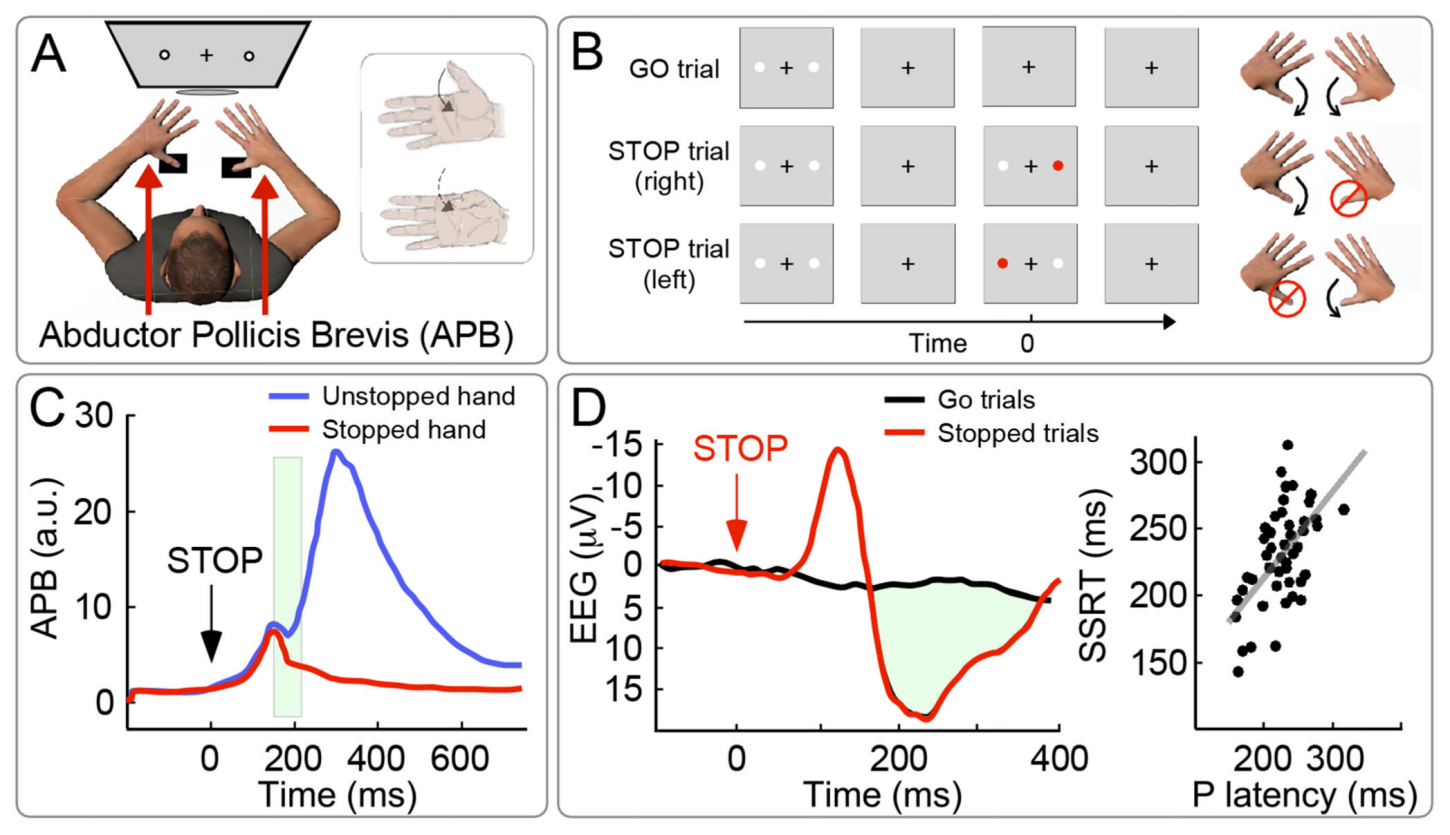
Action Stopping (AS). (A-B) On “go” trials, in response to visual cues, participants have to press two buttons, one with each thumb. On “stop” trials, an additional sudden visual stimulus prompts participants to stop the movement of one thumb but to continue the movement of the other thumb. Surface EMG is measured from the abductor pollicis brevis (APB) of both hands, i.e. a muscle subserving the thumb response. (C) The “stop” signal evokes a short-latency inhibition in the APB associated with the interruption of the thumb movement, starting ~150 ms following the “stop” signal (green shaded area). Notably, a transient inhibition at the same latency is also observed in the APB of the thumb completing the task without stopping. Adapted from (50). (D) The sudden “stop” signal also evokes a negative-positive potential in the scalp EEG. The latency of the EEG positivity correlates (between participants) with the stop signal reaction time (SSRT). Adapted from (69).

Surprisingly, nearly all studies using the SST have focused on estimating the stop signal reaction time, neglecting the modulation of the muscular activity before and during the actual stopping behaviour. Only a few recent studies have looked at this, using the following paradigm: on “go” trials, participants had to press two buttons in response to a visual cue, one with each hand. On “stop” trials, an additional (suddenly-appearing) visual stimulus prompted participants to suppress the response of one hand but to continue the response of the other hand [(50); Fig. 4A-B]. Surface EMG was measured from the *abductor pollicis brevis* of both hands, i.e. one of the muscles controlling the thumb, used for pressing the buttons. This paradigm allowed to sample EMG activity associated with both the stopped and the non-stopped action, and to compare those with the activity elicited by “go” trials. Two interesting observations were made. First, the EMG activity associated with successfully stopped actions displayed a short-latency inhibition, starting ~140 ms following the stop signal [(50); see also (51–53)]. This latency is compatible to (albeit slightly longer than^2^) the previously reviewed CMR, SLR and OMC. Second, a transient inhibition at the same latency was also present in the EMG measured from the other hand completing the task without stopping, implying that *all* ongoing actions were being stopped (Fig. 4C).

The latter observation is very important when we consider that, at first glance, the SST appears qualitatively different from the tasks used for measuring CMR, SLR or OMC. In particular, SST entails a voluntary response to a stimulus, while all previously described RABs are largely automatic responses, i.e. scarcely modulated by volition. However, AS is not strictly driven by volition either, because *all* ongoing actions are stopped, not only those that are intended to be stopped. In other words, AS has a “global” character [as reviewed in (54)]. This observation is in line with the recent proposal that AS is not merely proactive, but also reactive to the surprising nature of the stop signal [(55, 56) see also (57)]. In line with this hypothesis, it is well known that slower response times or even non-voluntary stopping of ongoing actions can follow abrupt unexpected events, i.e. in a fully reactive mode. This has been shown in psychophysical studies testing unexpected events such as action errors, unexpected action outcomes, or unexpected perceptual events (54, 58–62), using distinct sensory modalities such as audition (63), vision (64) and somatosensation (65). Notably, some of the above classes of stimuli are extremely similar to those optimally eliciting the previously discussed RABs. For instance, OMC are elicited by stimuli that entail unexpected action outcomes or action errors, while the SLR and the CMR are elicited by unexpected perceptual events.

The neural origin of AS has mostly been explored using EEG. Here, another interesting similarity with the other described RAB emerges: Just like the stimuli inducing the CMR, also the “stop” stimuli discussed here evoke a widespread negative-positive potential (Fig. 4D). Moreover, and again in line with the CMR, the latency of the evoked positive wave robustly predicts the stop signal reaction time (50, 54, 66–68).

## Concluding Remarks and Future Perspectives

The take-home message of this work is that a number of eye-opening similarities appear when the CMR, SLR, OMC and AS are critically compared. We have coined a unifying label for these phenomena – Reactive Adaptive Behaviour (RAB) – and defined four fundamental features that apply to all of them. These entail (i) the fast-rising nature of the RAB-evoking stimuli and, likewise, (ii) the fact that RAB occurs rapidly, within 150 ms following the stimuli. RAB is also (iii) adaptive, in that the behaviour is not stereotyped, but varies in response to the environmental context in a flexible manner that might ultimately enhance the efficiency of behaviour and, in the long term, survival. Finally, RAB is (iv) barely modulated by volition. A few additional similarities, albeit not yet conclusive, emerged. These are summarised in BOX 2, where we also suggest potentially fruitful pathways for future research.

These resemblances unavoidably trigger the question of whether all RABs have a common neural origin. Although we do not argue that all RABs rely on the very same neural structure, we do suggest that they likely share a common neural mechanism, perhaps working in synergy with RAB-specific cortical or subcortical structures. Such common mechanism is devoted to the rapid identification of important environmental events and the preparation of appropriate motor responses (10, 70–73). Note that several influential models of salience detection and orienting behaviour predict that salient events should have direct consequences on behaviour (74–77). Here we suggest that the RABs reviewed here (and possibly other similar behaviours) represent such consequences.

**Figure 5:**
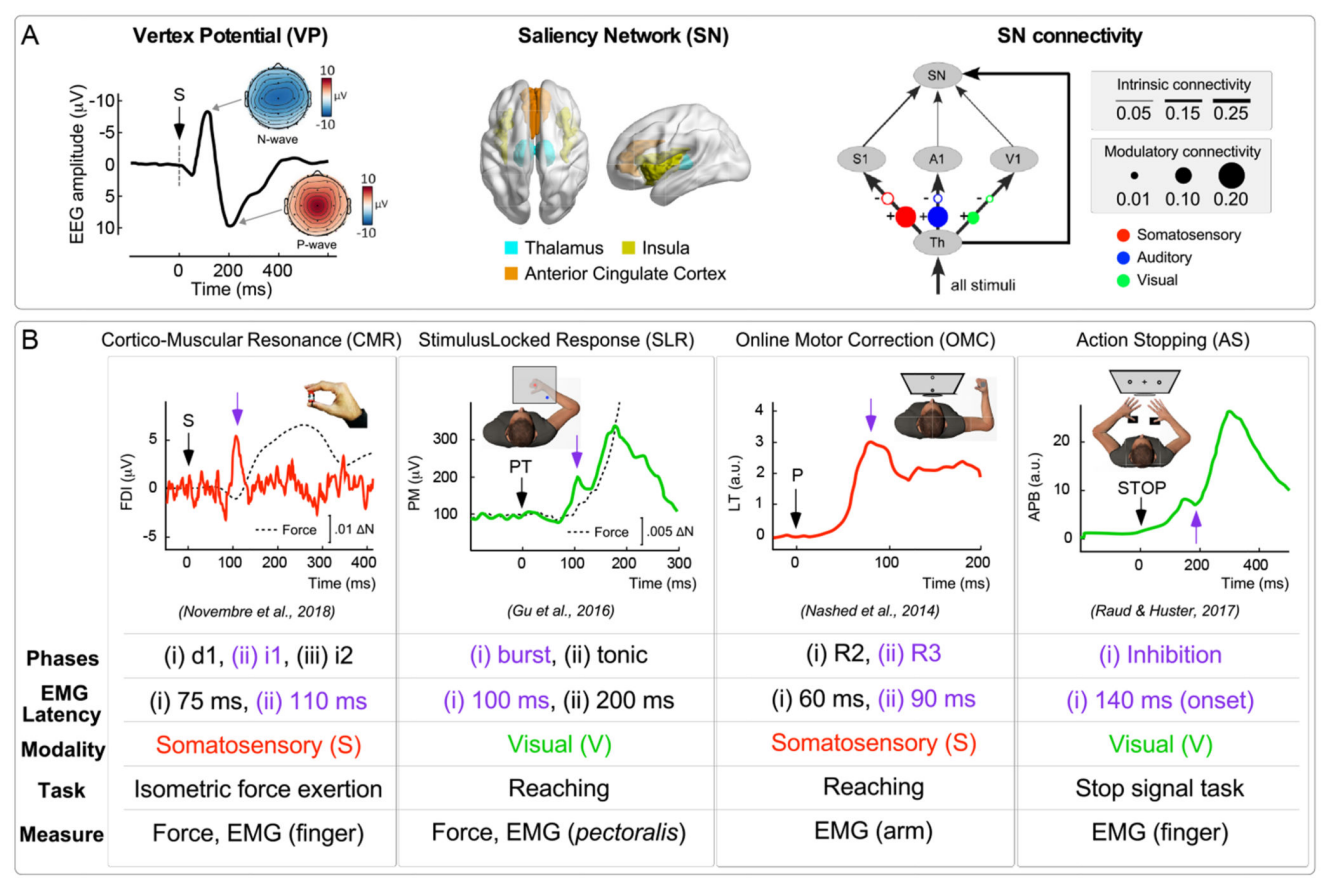
(A) Neural correlates of the Saliency Network (SN). Middle: the SN comprises the thalamus, the insula, and the anterior and middle cingulate cortex. Left: The SN activity manifests itself as a transient negative-positive electrocortical wave, maximal at the scalp vertex and therefore called vertex potential (VP). Right: Functional connectivity between the thalamus, the primary sensory cortices, and the cortical components of the SN (insular and cingulate cortex). The thickness of black lines line represents the strength of intrinsic connectivity. The size of colored dots/circles represents the strength of the modulatory effect exerted by external stimuli on each connection (colors represent stimulus modalities, plus (+) and minus (-) symbols represent enhancement and inhibition, respectively). Adapted from (72).(B) Illustration of all reviewed RABs, as characterized across four distinct studies (citations are embedded in the figure). Despite being elicited using clearly different tasks, all RABs entail an early, transient modulation of muscular activity at ~100 ms post-stimulus (purple arrow): the i1 of the CMR, the early burst of the SLRs, the R3 of the OMC and the inhibition of the AS. While we do not conclusively claim that these specific modulations are functionally equivalent, we stress that their slight differences in peak latencies can be explained by the sources of variability discussed in BOX 1. For instance, the longest latency of the AS response (discussed also in footnote 2) is consistent with the fact that it is evoked by visual stimuli (which are notoriously processed more slowly than somatosensory ones, see BOX 1) and measured in a distal muscle (the abductor pollicis brevis). Likewise, the shortest latency of the OMC response (in this example R3, but we could also consider R2) is compatible with the use of somatosensory stimuli eliciting it in a relatively proximal muscle (the lateral triceps).

One particular neural system that could be responsible for RAB is the Salience Network (SN), comprising the insula, the anterior cingulate cortex, the thalamus, and a number of other subcortical structures (70, 75) (Fig. 5A). The SN is known to be activated by salient events through rapid pathways that bypass primary sensory cortices (72), in order to swiftly guide and adjust behaviour, for instance via the anterior cingulate cortex that facilitates rapid access to the motor system. Remarkably, the functional properties of the SN are reminiscent of those characterizing RAB. For instance, the electrocortical SN activity (in particular the activity of the insula and the anterior cingulate cortex (10, 78–80)) manifests itself as a transient negative-positive wave, maximal at the scalp vertex and therefore called vertex potential (VP) (9, 10) (Fig. 5A). Alike RAB, the VP also occurs swiftly after abrupt or unexpected sensory stimuli, and, crucially, irrespectively of their sensory modality (an important aspect to consider given that the reviewed RABs are similarly elicited by stimuli belonging to distinct sensory modalities) (9, 10). Furthermore, the VP magnitude is not stereotyped, but very sensitive to contextual changes in the sensory scene (71, 81–83), a feature compatible with RAB’s adaptive character. Finally, the VP is a very robust and largely automatic response, poorly modulated by volition. For instance, the VP is elicited by salient stimuli also in unconscious individuals, e.g. during sleep (84), and its magnitude appears to be minimally affected by high-level cognitive expectations about the stimulus nature (81).

The contribution of a cortical network such as the SN might at a first glance appear difficult to reconcile with the rapidity of RAB. The reader might wonder whether short-latency motor responses like RAB are too fast to be integrated with sensory information processed at cortical level. However, decades of work in both physiology and psychology has recognized the existence of fast pathways allowing the human brain to quickly process and react to sudden and unexpected sensory information (76, 85). Abrupt salient stimuli – such as the ones triggering RAB – can activate the SN very rapidly (86), through extralemniscal, non-modality-specific parallel thalamocortical connections that by-pass primary sensory regions (72, 87) (Fig. 5A). This comes at the cost of degrading the fidelity of stimulus coding and the resulting perceptual processing (76, 85). However, this rapidity permits the human brain to swiftly execute actions (31), in particular when certain sensory events call for urgent behaviour, with no need for fine-grained perceptual processing. Such a prioritised, extremely fast pathway appears to be a good candidate mediating RAB.

The cortical origin of RAB, and its putative relationship with the SN, should be investigated pairing behavioural or muscular recordings with simultaneous measurements of electrocortical activity. When this was done (e.g. using EEG in CMR and some AS studies), the effect of sensory stimulation was studied not only on muscular activity and kinematics, but also on brain activity, thereby leading to a more comprehensive characterization of how the nervous system responds to salient changes in the environment (5, 6, 66, 67). Notably, these studies show that the trial-by-trial variability in VP amplitude or latency predicts the trial-by-trial variability of the RAB of interest. This fruitful approach, once applied to the entire range of RABs, will establish their relationship with the cortical SN, and thereby identify a possible common mechanism. In fact, although a similar approach has not yet been attempted with SLR and OMC, there is indirect evidence for such common mechanism. For instance, an enhanced VP (i.e. increased in amplitude and decreased in latencies) is evoked by visual stimuli having strong visual contrast (higher luminance) (88), just like SLRs do (23). Along the same line, the well-known hierarchical organization of ERPs across time – with increasingly complex computations reflected in longer-latency components (89) – is reminiscent of the progressively more complex mechanisms underlying OMC responses: while early R2 might reflect “how” to achieve the given goal, the late R3 might reflect “what” goal to achieve (3).

Having highlighted the remarkable similarities characterizing the above-reviewed RABs, we suggested to unify these phenomena proposing a common neural mechanism related to the detection and reaction of salient environmental events. We wishfully expect this effort to trigger curiosity and cross-field fertilization.

## BOX 1 Response latency and its sources of variability.

Several analogies across distinct RABs can be made on the basis of their response latency. Yet, caution must be exerted when doing so, considering some important physiological sources of latency variability. We discuss these below.

### Measure types

The earliest indices of peripheral motor activity are electromyographic (EMG) responses reflecting the electrical activity produced by skeletal muscles. These EMG responses are followed by changes in force, either isometric or entailing actual movements (i.e. kinematics), both having a considerable electromechanical delay (30-60 ms) with respect to their underlying EMG activity (90, 91). Even longer latencies are observed when RABs are studied measuring response times (RTs; such as pressing a button), as their latency reflects the final stage of a movement (25, 92).

### Sensory Modalities

The sensory modality of the stimuli used to elicit different RABs is another important factor to consider when comparing latencies across different studies. Cross-modal differences in both transduction and transmission times account for remarkable differences in response latency. It is difficult to precisely quantify these delays, as they also depend on the stimulus properties (see next point). Yet, using the peak latency of first negative wave of the supramodal and saliency-dependent EEG vertex potential as guidance (10), auditory stimuli yield the fastest responses, while responses to somatosensory stimuli delivered to the hand are slower by ~25 ms, and those to visual stimuli follow with an additional ~40 ms delay. This might contribute to some of the ostensive differences among RABs elicited by stimuli belonging to different sensory modalities.

### Stimulus properties

Response latency also depends on the physical properties of the sensory stimulus, such as its magnitude and rise-time (93, 94). This generalizes well across different sensory modalities (93). In addition, when eliciting RAB using somatosensory stimuli, it is also imperative to consider what body part is stimulated and, most importantly, what type of stimuli are delivered. Indeed, different somatosensory stimuli can activate entirely different receptor classes, associated with different nerve fibres having different axon diameters, hence conduction velocities (95, 96).

### Measured muscles

When measuring EMG (or related force/kinematic) activity, another factor causing differences in response latency is the proximity between the innervated muscle and the central nervous system. Considering the conduction velocity of both corticospinal tracts and motoneurons, the different travelling length can result in up to 20 ms in relatively extreme cases, e.g. when latencies of responses in the upper arm and the lower leg muscles are considered (97).

### Onset vs. Peak latency

Another factor significantly affecting latency comparison is whether the onset or the peak of a response is measured. While peak latency is relatively straightforward to calculate (at least when dealing with transient responses), onset latency is more difficult to measure, and it is often estimated computing the response’s first-order derivative. Although onset and peak latencies are clearly correlated with one another, they sometimes have different predictive power (69). For instance, onset latencies can give more reliable estimates of neurophysiological properties such as conduction velocities of peripheral afferents (98, 99).

### RAB-related neural measures

Just as the various measures of motor activity discussed above (EMG, force, RT), also the indices of stimulus-evoked neural activity can have different temporal profiles and considerable latency variability. Invasive recordings such as single and multi neuron activity sometimes yield earlier latencies compared to local field potentials (100). In turn, the EEG signal, which is recorded non-invasively from the skull, often depends on the simultaneous activity of multiple generators and therefore can display significant latency differences compared to local field potentials (80, 101). When examining the relationship between motor and neural measures these sources of latency variability should also be taken into account.

## BOX 2 Outstanding Questions

Having highlighted a number of features shared across all RABs, we outline some research directions that we consider important to identify a putative shared neural mechanism.

1. *Latencies*. All reviewed RABs entail a transient modulation of muscular activity at ~100 ms following the stimulus (Fig. 5B). Does this similarity speak in favour of a shared neural mechanism? To address this question appropriately, future research will need to control for the different physiological parameters that are known to impact upon the latency of a muscular response (BOX 1). Should different RABs be subserved by a shared neural mechanism, then controlling for these sources of variability will permit to observe quasi-identical response latencies in each of them.
2. *Multiple phases*. CMR, SLR and OMC are associated with muscular responses entailing multiple phases. Instead, the two studies characterizing EMG during AS have described only one (inhibitory) phase (50, 51). Yet, it should be noted that recent work on rodents has suggested that AS might be achieved by the combination of two consecutive neural inhibitory processes: an early one only pausing the action, and a later one potentially cancelling it (102, 103). Future work should investigate the hypothesis of a multi-phasic AS process examining humans’ muscular activity, particularly considering the interesting analogies between AS and other RABs (104).
3. *Supramodality*. CMR, OMC and AS can be elicited by stimuli belonging to more than one sensory modality. Whether this is also the case in SLR (so far elicited only by visual stimuli) has never been explicitly tested, although a recent study showed that by pairing the SLR-evoking visual stimulus with somatosensory or auditory stimuli facilitates the SLR (105). Demonstrating that SLRs can in fact be evoked by non-visual stimuli would provide additional evidence in favour of a shared neural mechanism responsible for all RABs.
4. *Active cortico-spinal drive*. All RABs are typically or preferentially observed when the cortico-spinal drive is tonically active, i.e. while a participant executes an action or exerts a constant isometric force. While this feature can potentially link all RABs within a unifying neural mechanism, demonstrating that only some (but not all) RABs could be evoked in the absence of an active cortico-spinal drive would instead speak against such hypothesis.
5. *Relationship with EEG signals*. CMR and AS, when recorded simultaneously to the EEG, reveal to be tightly coupled to both event related potentials (5, 69) and neural oscillations (6, 106). Demonstrating a similar coupling in SLR or OMC would provide additional evidence in favour of a shared neural mechanism.
6. *Inter-individual variability*. A promising avenue of research pointing towards a shared neural mechanism could be comparing inter-individual variability in response magnitude across different RABs. Should the same neural mechanism be responsible for all RABs, then inter-individual variability should be similar across RABs (notably, this approach would require careful control of several parameters such as e.g. stimulus saliency or activity of the corticospinal drive).

## Highlights

- Reactive Adaptive Behaviour (RAB) consists of short-latency motor responses to sudden sensory stimuli.
- RAB is flexible and largely independent of volition.
- We review four independent research fields, progressing in parallel and each characterizing a putatively distinct RAB.
- We highlight remarkable similarities across the reviewed RABs.
- We suggest that the reviewed RABs could be subserved by the same fundamental neural mechanism.

## Footnotes

1 The fact that OMC is poorly modulated by volition does not imply that this behaviour would not be consistent with what an individual would intentionally do in a given circumstance. Rather, it simply implies that this behaviour is triggered automatically by the stimulus without an individual deliberately choosing to act. Notably, according to some computational accounts such as the “optimal feedback control”, relatively complex goal-directed behaviours can be automatically produced following a sophisticated manipulation of sensory feedback (36, 107). From this perspective, OMC and voluntary actions could lead to the very same behaviour, being it subject to volition or not.

2 The latency of the EMG modulation associated with AS is relatively longer than the earliest EMG modulations associated with the RABs discussed so far (see Fig. 5). This apparent inconsistence might be explained by the fact that, in the SST, two distinct motor processes - the excitatory “go” and the inhibitory “stop” – compete with each other (Fig. 4B) (47). Importantly, because the “go” process is triggered before the “stop” process, the latter must override the former in order to successfully stop the ongoing action, which likely has a cost in latency. We have run preliminary simulations supporting this point.
